# Concentrated Growth Factors as an Ingenious Biomaterial in Regeneration of Bony Defects after Periapical Surgery: A Report of Two Cases

**DOI:** 10.1155/2019/7046203

**Published:** 2019-01-22

**Authors:** Nivedhitha Malli Sureshbabu, Kathiravan Selvarasu, Jayanth Kumar V, Mahalakshmi Nandakumar, Deepak Selvam

**Affiliations:** ^1^Department of Conservative Dentistry and Endodontics, Saveetha Dental College, Saveetha Institute of Medical and Technical Sciences, Saveetha University, 162, Poonamallee High Road, Chennai, 600077 Tamil Nadu, India; ^2^Department of Oral and Maxillofacial Surgery, Saveetha Dental College, Saveetha Institute of Medical and Technical Sciences, Saveetha University, 162, Poonamallee High Road, Chennai, 600077 Tamil Nadu, India; ^3^Department of Oral Medicine and Radiology, Saveetha Dental College, Saveetha Institute of Medical and Technical Sciences, Saveetha University, 162, Poonamallee High Road, Chennai, 600077 Tamil Nadu, India

## Abstract

The overall success of a periapical surgery is assessed in terms of regeneration of functional periradicular tissues. The regenerative potential of platelets has been well documented. This article describes the use of concentrated growth factors (CGF), a new family of autologous platelet concentrates, as a sole material for bone regeneration after periapical surgery. 32- and 35-year-old female patients diagnosed with Ellis Class IV, an open apex in 11 with apical periodontitis in 11 and 12 and previously root canal-treated 31 and 41 with a chronic apical abscess, respectively, were managed with endodontic surgery. Subsequent to apicectomy and retrograde filling, the CGF fibrin block and membrane were used before suturing. There was uneventful healing during the immediate post-op and the subsequent follow-up periods. CGF is produced by a differential centrifugation process that results in the formation of a denser fibrin matrix richer in growth factors than those observed in PRF. Reasonable osseous healing was seen as early as 6-month follow-up, thereby recommending the use of CGF as an alternative to bone grafts and membranes in extensive periapical lesions to enhance bone regeneration and to decrease the healing time.

## 1. Introduction

Periapical surgery is the treatment of choice for teeth with persistent apical periodontitis when the conventional nonsurgical method fails to achieve the principal objectives [1]. It has been predicted that the smaller defect heals around one year, whereas for the larger defects, the healing time may be more than two years [2]. However, recently, Lin et al. have demonstrated that the healing of periapical tissues is a “programmed event.” More than the size of the lesion, it is the microenvironment consisting of the progenitor/stem cells, extracellular matrix, and bioactive molecules that plays a crucial role in tissue regeneration or scar formation during wound healing [3].

Platelet-rich plasma (PRP) and plasma rich in growth factors (PRGF) classified as the first generation of autologous platelet concentrates (APCs) have demonstrated promising results in the management of extensive periapical lesions, apicomarginal defects, and combined endo-perio lesions and also improved the quality of life of patients undergoing endodontic surgery [4]. However, both have several drawbacks like use of anticoagulant, artificial polymerization, and a time-consuming two-step centrifugation process.

Platelet-rich fibrin (PRF), considered as the second generation platelet concentrate and classified into L-PRF, A-PRF, i-PRF, CGF, PRFM, and Vivostat PRF [5]. A systematic review reported that APCs have a favourable effect on patients' quality of life and are supportive for bone regeneration after endodontic surgery [6].

Concentrated growth factor (CGF) is a newer second generation platelet concentrate that is prepared by centrifuging blood samples at alternating and controlled speeds using a special centrifuge. Differential centrifugation results in the formation of a denser fibrin matrix richer in growth factors than those observed in the PRF and PRP. To our knowledge, this is the first case report for the use of CGF alone in endodontic surgery. For describing the case reports, we have followed the checklist given in CARE guidelines (http://www.care-statement.org/) [7].

## 2. Case Presentation

### 2.1. Case Report 1

A 32-year-old female patient was referred to the Department of Conservative Dentistry and Endodontics with chief complaint of discolouration of the upper right front tooth (tooth 11) for the past 6 months. Past dental history revealed a traumatic fall 5 years ago which involved the upper front teeth for which no dental treatment was sought for. Recently, as the discolouration gradually worsened, the patient sought dental treatment to restore esthetics.

**Figure 1:**
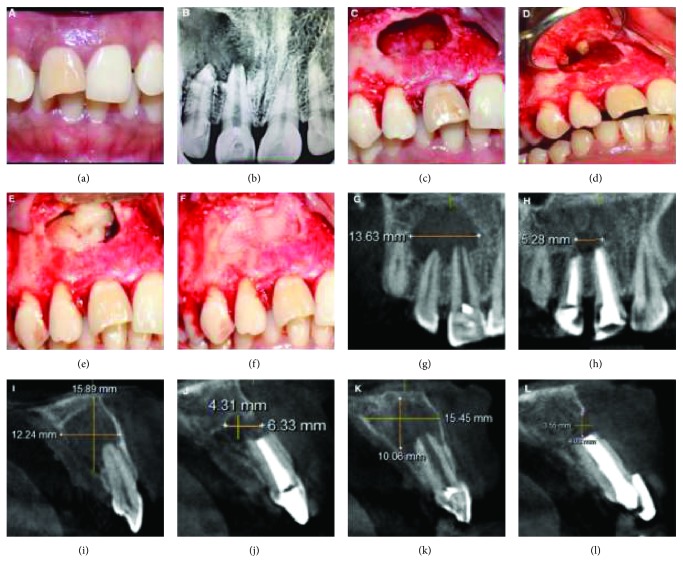
Preoperative clinical and surgical procedure and radiographic images: IOPA and CBCT images (Case Report 1). (a) Pre-op clinical photograph showing discoloured 11 with chipping of the incisal edge of the crown. (b) Preoperative IOPA showing the periapical lesion involving the apices of 11 and 12 and extending to the mesial margin of 13. (c) Complete curettage of the lesion and apicectomy done in 12. (d) Retrograde preparation and filling with MTA in 11 and 12. (e) CGF fibrin block placed in the bony cavity. (f) CGF membrane placed to cover the bone defect. (g) Pre-op CBCT image showing the lesion measurement in the coronal slice. (h) Post-op CBCT image showing the lesion measurement in the coronal slice. (i) Pre-op CBCT image showing the lesion measurement in sagittal slice corresponding to 12. (j) Post-op CBCT image showing the lesion measurement in sagittal slice corresponding to 12. (k) Pre-op CBCT image showing the lesion measurement in sagittal slice corresponding to 11. (l) Post-op CBCT image showing the lesion measurement in sagittal slice corresponding to 11.

Intraoral clinical examination revealed a slightly discoloured 11 and chipping of the incisal edge with no presenting symptoms (Figure 1(a)). Both 11 and 12 responded negatively to the electric pulp tester (Gentle Pulse™ Pulp Vitality Tester, Parkell, USA) and elicited mild tenderness on percussion.

On radiographic examination (VistaScan Mini, UK), extensive periapical radiolucency involving the roots of 11 and 12 and an open apex of 11 was revealed (Figure 1(b)). CBCT for this case (Dentsply Sirona, Orthophos XG 3D) was taken at standardized settings (90 kV, 6 mA, 5 × 5.5 cm, 160 *μ*m, and 14 s) to assess the exact location, size, and extent and proximity of the lesion to anatomical structures.

The preoperative measurements of the lesion as seen in CBCT in different planes can be seen in Figures 1(g), 1(i), 1(k). According to the CBCT-PAI scores, it was graded as a 5D lesion [8]. The score 5 indicates that the diameter of the periapical lesion is greater than 8 mm, and D represents destruction of the periapical cortical bone in the palatal region.

A tentative diagnosis of an Ellis Class IV fracture and open apex in 11 with apical periodontitis in 11 and 12 was made. The differential diagnosis could be a chronic periapical abscess, periapical cyst, and periapical granuloma. The treatment plan was root canal treatment for 11 and 12. The patient was informed about the risks and benefits of the procedure and a written consent was taken.

Under local anaesthesia (2% lignocaine in 1 : 200,000 dilution adrenaline, Neon Laboratories Ltd.) and rubber dam isolation, an access cavity was prepared in 11 and 12 with an Endo access bur (Dentsply Maillefer, Switzerland). The working length was determined with an apex locator (Propex Pixi, Dentsply Maillefer, Switzerland) and confirmed with radiograph. Cleaning and shaping was initiated with 45 K file (Mani, Inc. Japan), and apical preparation was performed till size 80 K file in 11. In 12, apical preparation was done till size 45 K file to full working length, after which step-back preparation was done till 80 K file. Routine root canal-shaping procedure was done along with copious irrigation using 3% sodium hypochlorite (VIP, Vensons, India) and final flush with 0.9% physiologic saline (acuLIFE, India). Calcium hydroxide medicament (RC Cal, Prime Dental Products, India) was placed thrice for a period of 1 week each. As the root canals exhibited persistent discharge of exudates and due to two prognostic factors (size of the lesion which was more than 10 mm and the thinning or destruction of the palatal bone) which were unfavourable for this case [9], periapical surgery was planned following the recommendation given by the Spanish Society of Oral Surgery (point 3: a radiotransparent lesion measuring over 8 to 10 mm in diameter).

The root canals were obturated a day before the surgery using custom-made roll cone technique for 11 and conventional cold lateral compaction technique for 12.

During surgery, after achieving adequate anaesthesia, a crevicular incision was placed from 22 to 14 with a vertical releasing incision on the mesial aspect of 14 to reflect a full thickness mucoperiosteal triangular flap. Cortical softening of the periapical bone was noted from regions 11 to 13. A bony window was created and thorough curettage was done (Figure 1(c)).

Apicoectomy was performed extending 3 mm into the canal space using no.702 tapered fissure burs (SS White burs) in 12. Apicoectomy was not done in 11 because of the presence of an open apex. Root end cavity preparation was done using zirconium nitride ultrasonic retro-tips (Dentsply Maillefer, Switzerland) in 11 and 12. Subsequently, retrograde filling was done with Mineral Trioxide Aggregate (MTA Angelus® Brazil) (Figure 1(d)). Later, the surgical site was prepared for placement of CGF fibrin gel and CGF membrane.

A standard, disposable, two 10 mL nonanticoagulant glass tubes and a matching centrifuge device (MEDIFUGE, Silfradent s.r.l., S. Sofia, Italy) were used. 20 mL of intravenous blood sample from the patient was placed in centrifuge tubes without anticoagulants and accelerated for 30 s, centrifuged at 2700 rpm for 2 min, 2400 rpm for 4 min, 2700 rpm for 4 min, and 3000 rpm for 3 min, and decelerated for 36 s to stop. All of these processes are adjusted automatically by “preprogramming” in the machine.

From the three layers formed, the uppermost platelet-deprived fraction was removed with a sterile syringe. The layer in the form of a fibrin gel containing the CGF was separated from the red blood cell layer. The prepared CGF fibrin gel was placed inside the surgical site and covered with CGF membrane. The layer in the form of a membrane containing the concentrated growth membrane was held with a hemostatic clamp and separated from the RBC layer by using microsurgical scissors. The CGF layer is then placed in a condensing disc and compressed to convert to CGF membrane. (Figures 1(e), 1(f)).

Subsequent to CGF placement, the flap was closed with 3-0 vicryl sutures (Ethicon Inc. Piscataway, USA). Postoperative instructions were given and systemic antibiotics, analgesics, and supplemental 0.2% chlorhexidine mouthwash were prescribed.

The postoperative CBCT at 1-year follow-up showed satisfactory healing with evident reduction in lesion size as shown in Figures 1(h), 1(j), 1(l), and the patient was asymptomatic at all the recall periods, suggesting a successful treatment outcome. The patient is kept under review, to be followed up after 18 months, 24 months, and 36 months.

### 2.2. Case Report 2

A 35-year-old female patient reported mild swelling and pus discharge in the lower front region of the mouth for the past 2 months. Past dental history revealed trauma to lower anterior teeth 4 years ago, following which she underwent endodontic treatment. History suggested that there were multiple retreatments in the past for the current chief complaint.

**Figure 2:**
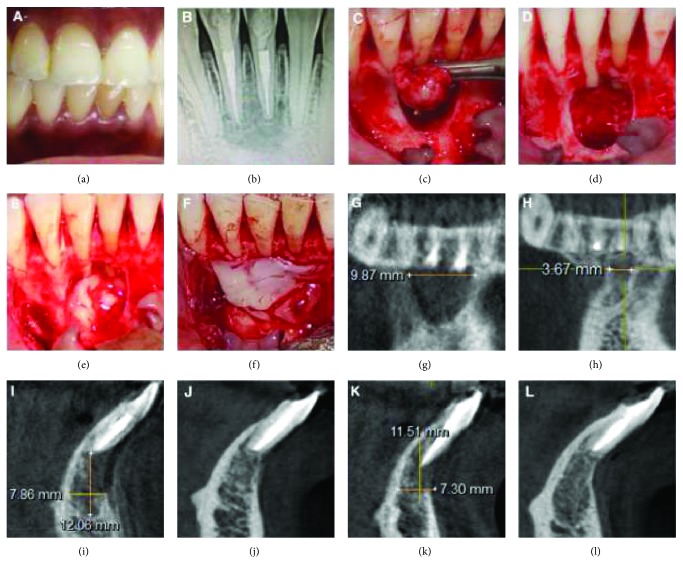
Preoperative clinical and surgical procedure and radiographic images: IOPA and CBCT images (Case Report 2). (a) Pre-op clinical photograph shows discoloured 31. (b) Preoperative IOPA showing the periapical lesion involving the apices of 11 and 12 and extending to the mesial margin of 13. (c) Complete curettage of the lesion. (d) Apicectomy done and MTA retrograde filling given in 31 and 41. (e) CGF fibrin-rich block placed in the bony defect. (f) CGF membrane placed before suturing of the flap. (g) Pre-op CBCT image showing the lesion measurement in the coronal slice. (h) Post-op CBCT image showing the lesion measurement in coronal slice. (i) Pre-op CBCT image showing the lesion measurement in sagittal slice corresponding to 41. (j) Post-op CBCT image showing the lesion measurement in sagittal slice corresponding to 41. (k) Pre-op CBCT image showing the lesion measurement in sagittal slice corresponding to 31. (l) Post-op CBCT image showing the lesion measurement in sagittal slice corresponding to 31.

On clinical examination, 31 was discoloured with mild swelling on the labial aspect (Figure 2(a)). Radiographic examination revealed a well-obturated 31 and 41 with large periapical radiolucency (Figure 2(b)). The CBCT-PAI score was 5D (Figures 2(g), 2(i), 2(k)). Based on the above findings, a diagnosis of previously root canal-treated 31 and 41 with a chronic apical abscess was made. History suggested that there were multiple retreatments in the past for the current chief complaint, with presenting complaint of recurring, intermittent swelling with pus discharge in the lower front region. And radiographic examination revealed adequate obturation and apical seal, with nonhealing chronic periapical radiolucency. The treatment plan of periapical surgery was decided in accordance with the indications given by the European Society of Endodontology, 2006 (point 3: presence of persisting periapical disease after root canal retreatment).

Under local anaesthesia, the full thickness trapezoidal mucoperiosteal flap was reflected with vertical releasing incisions taken from the mesial aspect of 43 and 33. The surgical site was flushed with sterile saline after thorough curettage of the lesion (Figure 2(c)). Following which, apicectomy and retrograde MTA filling were done (Figure 2(d)). The CGF preparation was similar as described in Case Report 1, and the prepared CGF was placed inside the surgical site and covered with CGF membrane (Figures 2(e), 2(f)). The flap was approximated with interrupted sutures. Follow-up CBCT at 1 year revealed complete healing with complete bone repair, evidently seen in coronal and sagittal views (Figures 2(h), 2(j), 2(l)).

At 1-year follow-up with CBCT, livewire segmentation using OSIRIX Version 9.5 (PIXMEO, Geneva, Switzerland) was done to delineate the lesion from the healthy bone. In case 1, the preoperative and postoperative volume calculations were 0.7862 cm^3^ and 0.08 cm^3^, respectively. The lesion size reduction was found to be 89.2%. In case 2, the preoperative and postoperative volume calculations were 0.1358 cm^3^ and 0.0101 cm^3^, respectively. The lesion size reduction was found to be 92.5%.

## 3. Discussion

Our case reports are unique in that CGF was produced using the recommended centrifuge, and it was used as a sole material to understand its exclusive role in repair and regeneration after periapical surgery.

During the preparation of APCs, the quality of the resultant product is greatly influenced by centrifugal characteristics like rotational speed (rpm), time (min), and centrifugation protocols. The centrifugation method for CGF is “preprogrammed” and the resultant CGF is stronger, thicker, and abundant with growth factors. The centrifuge is equipped with self-ventilation that prevents the temperature rise which helps to maintain the viability of enmeshed cells in the fibrin matrix. The dense three-dimensional network of the fibrin ensures slow release of growth factors. Park et al. affirmed that CGF consisted of thicker fibrinogen fibres per area unit and regular fibrinogen structures compared to PRF [10].

The presence of growth factors and leucocytes including also the few CD34+ circulating cells that are concentrated in a small volume is the probable reason for this success, since all the elements have been demonstrated to play an important role in vascular maintenance, angiogenesis, and neovascularization. Park et al. reported that TGF-*β*1 was released for one week whereas PDGF-BB for 3 weeks and identified that VEGF was one and a half times more in CGF than PRF [10]. However, Qin et al. showed that TGF-*β*1 had a slow release for 13 days [11]. In a contrasting study, the levels of growth factors released (except bFGF) did not differ significantly among PRP, PRF, and CGF [12].

Growth factors act on target tissues and regulate a variety of cellular events including cell migration, proliferation, and differentiation. Borsani et al. have ascertained that CGF addition enhanced cell proliferation of fibroblasts, endothelial cells, and osteoblasts which are involved in angiogenesis, tissue remodelling and regeneration [13].

There are two case reports in the literature which describe the utilization of CGF in periapical surgery. In the first, CGF was not prepared with the special centrifuge [14]. In the second, CGF was used along with the sticky bone and Mphi laser [15]. Even though both the cases had unfavourable prognostic factors, the follow-up has affirmed the encouraging effect of CGF which has shortened the healing time of extensive periapical lesions to 6 months. This finding corroborates with the outcome of the case reports by Sohn which described two cases of sinus augmentation in which CGF alone was used as a substitute for bone graft and the results established that healing was reduced to half the average healing period without bone graft placement [16]. The benefits of CGF are that it is autologous, can be easily prepared, and cost-effective than bone grafts and membranes in extensive periapical lesions. Taking into account the above benefits, CGF can be earmarked as an ingenious biomaterial for bone regeneration.

## 4. Conclusion

Considering the encouraging result of these case reports, concentrated growth factors could be recommended as an alternative to bone grafts and membranes in extensive periapical lesions to enhance bone regeneration and to decrease the healing time. However, this case series is an initiative for application of CGF in endodontic surgery. The utilization of CGF in endodontics can be extended to revascularization procedures and endo-perio lesions also. However, the major limitation is that according to the CEBM level of evidence, the case reports have the lowest level of evidence. A well-designed randomized clinical trial is recommended to comprehend the long-term risks and benefits of using CGF in regenerative endodontics.
